# Attitudes of U.S. Hispanic and non-Hispanic women toward congenital CMV prevention behaviors: a cross sectional study

**DOI:** 10.1186/s12884-018-1807-0

**Published:** 2018-05-24

**Authors:** Rosemary Thackeray, Brianna M. Magnusson, Erica Bennion, Natalia N. Nielsen, Ryan J. Bailey

**Affiliations:** 1 Department of Health Science, Brigham Young University, Provo, UT USA; 2 Department of Epidemiology and Biostatistics, University of South Florida, Tampa, FL USA

**Keywords:** Cytomegalovirus, Attitudes, Hispanic, Prevention, Subjective norms, Women

## Abstract

**Background:**

Congenital cytomegalovirus (CMV) infection is the most common intrauterine infection. The only way to protect against congenital CMV infection is to practice CMV prevention behaviors. CMV seroprevalence rates are high in Hispanic women. It is unknown whether communication strategies should differ by ethnicity. The purpose of this study was to understand differences between U.S. Hispanic and non-Hispanic women’s attitudes toward CMV prevention behaviors and examine the relationship between perceived subjective norms and these attitudes.

**Methods:**

This was a cross-sectional study using an online panel. Participants were U.S. women of childbearing age. The dependent variable was attitude toward practicing CMV prevention behaviors, specifically avoiding sharing cups, food, and utensils with a child and not kissing a child on the lips.

**Results:**

Among 818 women (50% Hispanic), 16.8% of Hispanic women and 9.7% of non-Hispanic women (*p* = 0.002) reported familiarity with CMV. Attitudes toward CMV prevention through avoiding sharing behaviors (*M*_*Hispanic*_ = 5.55 vs. *M*_*non-Hispanic*_ *=* 5.20; *p* = 0.002) and not kissing a child on the lips (*M*_*Hispanic*_ = 4.80 vs. *M*_*non-Hispanic*_ = 4.21; *p* = 0.001) were positive for both ethnicities, but higher for Hispanic women. Hispanic women (*M* = 5.11) reported higher perceived behavioral control for avoiding kissing a child on the lips than non-Hispanic women (*M* = 4.63; *p* = 0.001). Hispanic women who were U.S. born or spoke English primarily more frequently kissed a child on the lips or engaged in sharing behaviors. Additionally, those who spoke Spanish mostly held more positive attitudes toward not kissing on the lips. Significant predictors for more positive attitudes toward CMV prevention behaviors were associated with perceived subjective norms, perceived behavioral control and pre-survey participation in risk behaviors.

**Conclusions:**

Hispanic women have more positive attitudes toward CMV prevention behaviors than non-Hispanic women, however in regression models other factors are more important predictors of positive attitudes than ethnicity. In developing strategies to encourage women to practice CMV prevention behaviors, a focus on further understanding and increasing subjective norms and perceived control over those behaviors may be warranted.

**Electronic supplementary material:**

The online version of this article (10.1186/s12884-018-1807-0) contains supplementary material, which is available to authorized users.

## Background

Cytomegalovirus (CMV) is a prevalent virus, affecting people of all ages. One-third of U.S. children are CMV infected by age 5 and > 50% of adults become infected by age 40 [1]. Following primary infection, reactivation or reinfection may occur. For the majority of healthy persons, CMV results in asymptomatic or non-specific infection of mild severity and short duration [2]. However, CMV contracted or reactivated during the pre- or peri-conception periods or during pregnancy can result in congenital CMV infection [3].

Congenital CMV infection is the most common intrauterine infection with an estimated birth prevalence of 0.64% [4]. An estimated 13% of CMV infected infants are born with apparent illness [5]. Approximately half (40–58%) of surviving symptomatic and 13.5% of asymptomatic infants experience long-term sequelae [5]. Complications of congenital CMV infection include hearing loss, vision loss, premature birth, damage to the internal organs, growth restriction, microcephaly, intellectual disability, poor motor control, and death [5].

CMV is transmitted through contact with bodily fluids including urine and saliva. Infection during pregnancy is most likely to occur through sexual contact or contact with young children [6]. Young children shed the virus more abundantly and for longer periods than do infected adults [7–9]. As such, contact with young children’s urine or saliva increases the risk of transmission. A quarter of parents of young children with CMV viral shedding seroconvert within a year [7].

To date, no licensed CMV vaccine is available. As such, behavioral modification is the only intervention available to reduce pregnant women’s exposure to CMV and thus reduce the risk of babies being born with CMV. Recommended behavioral changes focus on limiting contact with children’s urine or saliva by avoiding sharing cups, utensils, or food with children, washing hands following diaper changes, and not putting a child’s pacifier in your mouth [10]. Evidence suggests that these behaviors may result in significant reduction in CMV seroconversion rates [11].

U.S. Hispanics ages 6–49 years have nearly twice the IgG seroprevalence for CMV compared to non-Hispanic whites (76.9% vs. 39.5%) [12]. Similarly, 31% of U.S. Hispanic children aged 1–5 years compared to 11% of non-Hispanic white children are seropositive for CMV IgG [12]. These demographic differences in CMV prevalence suggest the need for CMV prevention messages targeting higher risk persons [13]. However, none of the extant literature has examined differences between demographic groups that could inform communication.

The primary purpose of this study was to understand U.S. Hispanic women’s attitudes toward CMV prevention behaviors and how these attitudes may differ those of non-Hispanic women. Additionally, we examined the relationship between two additional variables that, along with attitudes, are theorized to predict behavioral intention: subjective norms and perceived behavioral control [14, 15]. Subjective norms reflect whether friends, family and peers approve or disapprove of a behavior [14]. Perceived behavioral control represents the degree to which the person believes they have the ability to take action to perform the behavior [14]. We also examined the influence of past frequency of engaging in CMV prevention behaviors. Because Hispanic seroprevalance is high, it is thought that Hispanics may engage in these behaviors at different rates than non-Hispanic women. Additionally, a person’s background experience, in this case, frequency of practicing the prevention behaviors, may interact with subjective norms, perceived behavioral control and attitudes to influence intention [14].

Prior research indicates that the majority of women regularly perform hand hygiene and few women regularly put a child’s pacifier in their mouth [16, 17]. For this reason, this paper focuses on the less practiced CMV prevention behaviors of not sharing food, cups, and utensils with children and not kissing children on the lips.

## Methods

### Study design and participants

This cross-sectional study included U.S. Hispanic and non-Hispanic women 18–40 years of age, with a child ≤5 years of age living at home. Women who had ever worked as a health care provider or had a child with a diagnosed disability were excluded as both were hypothesized to have CMV knowledge that differ from those of the general population. Research participants were existing members of Survey Sampling International’s (SSI) Hispanic or standard market research online panel. SSI randomly selected participants who, based on their panel profile, were highly likely to qualify for participation in the study. Completion of the survey indicated their consent. The Brigham Young University institutional review board approved the study. The survey was conducted in English. SSI indicated that all participants join their panel and complete surveys in English. To estimate differences in the proportion of women who know about CMV we needed a sample size of 350 in each group [18]. This calculation was based on a desired precision of 5% and previous research that indicated the proportion of women who knew about CMV was 12%. This also provided adequate sample to compare means in attitudes scores between the two groups.

### Instrumentation

The dependent variable was attitude toward practicing the CMV prevention behaviors, specifically avoiding sharing cups, food, and utensils with a child and not kissing a child on the lips. Attitude was assessed using a seven-point semantic differential scale with four different descriptors: impractical-practical, inconvenient-convenient, difficult-easy, unrealistic-realistic [17]. Ratings were averaged over the four scales to create a single attitude score for each CMV prevention behavior ranging from 1 to 7 with higher scores indicating more positive attitudes toward performing that behavior.

Independent variables included demographics, background knowledge of CMV, past participation in CMV prevention behaviors, parental affection, perceived behavioral control, and subjective norms.

Demographic questions included educational level, annual household income, race, ethnicity, marital status, employment status, health insurance, pregnancy status, number of children at home, and household crowding index. Crowding was categorized as high (> 1 person/room) or average/low (< 1 person/room) [19]. Average and low were combined as few people (*n* = 53) in the sample met the definition of low crowding. Household income was converted into percentage of the federal poverty level (FPL) using household size and categorized as < 100% FPL, 100–200%FPL, and > 200% FPL [20]. Two questions served as proxy measures for acculturation for Hispanic respondents: country of birth and the language spoken most often at home.

CMV background knowledge was measured by 11 items [16]. Response options were true, false and I don’t know. These items were coded as 1 if correct and 0 otherwise and summed to create a knowledge score (Cronbach’s alpha 0.92). Participation in behaviors that increase CMV risk was measured by asking about the frequency of engaging in four behaviors: sharing food, cups and utensils and kissing children on the lips [16].

Perceived behavioral control was measured by two items adapted from Yardley, Miller, Schlotz, and Little [21]. The questions asked about their *confidence* that they could perform the prevention behaviors and if it was *possible* to perform these same behaviors. A combined perceived behavioral control score for avoiding the three sharing behaviors (Cronbach’s alpha = 0.92) was created as a separate score for avoiding kissing a child on the lips (Cronbach’s alpha = 0.89).

Parental affection was measured with items adapted from the Parenting Styles and Dimensions Questionnaire [22]. Each of the three items used the question stem “I express affection by…” and inquired about the frequency of “hugging”, “kissing” or “holding” their child. Responses were on a 5-point scale. Few women responded “never” or “once in a while” to these questions so the variable was recoded into three categories: always, very often and not often.

Subjective norms toward the CMV prevention behaviors were measured by four questions adapted from Steele and Porche [23]. Respondents were asked to rate their level of agreement that most people who are important to them or whose opinion they value a) practice the target behaviors and b) think that she should engage in the target behaviors. During questionnaire pre-testing, we learned that people who are important to them or whose opinion they value are not limited to women rearing children and therefore they are not practicing the CMV prevention behaviors. Thus, after pre-testing we eliminated that aspect of the standard normative belief questions. We calculated a subject norms score for the three sharing behaviors (Cronbach α = 0.95) and beliefs toward kissing a child on the lips (Cronbach α = 0.89).

### Statistical analysis

Frequencies and proportions for categorical variables were calculated to describe the demographic characteristics of the sample. Statistical differences between Hispanic and non-Hispanic women were determined using a chi-square test for the difference in proportions. Means and 95% confidence intervals were calculated for frequency of participation in CMV risk behaviors, attitudes scales, perceived behavioral control, and subjective norms scales. T-test and ANOVA were used to evaluate differences in means between subgroups of Hispanic women (those born in the U.S. and the primary language spoken at home) and between Hispanic and non-Hispanic women.

Factor analysis confirmed findings from previous work [17] which indicated that attitudes toward CMV prevention behaviors cluster by behavior type. Thus, women’s attitudes toward the CMV prevention behaviors were assessed using average scores from semantic-differential scales for the collective sharing behaviors (12 items) and for kissing behavior (4 items).

Linear regression was used to examine associations between demographic and behavioral variables. Two models (one each for sharing and kissing) were created for the primary outcome of attitudes toward CMV prevention behaviors. As we hypothesized that perceived behavioral control and frequency of performing CMV risk behaviors would be important predictors of attitudes, we also constructed regression models to assess predictors of these constructs for each behavior group. All six regression models included the following independent variables: ethnicity, age of youngest child at home, maternal age and education level, marital status, level of household crowding, whether the respondent was pregnant or planning a pregnancy, familiarity with CMV, subjective norms and parental affection. Models assessing perceived behavioral control also controlled for frequency of performing CMV risk behaviors. Models assessing attitudes also controlled for frequency of performing CMV risk behaviors and perceived behavioral control. Models were constructed using manual backward elimination. Covariates that did not reach significance at α = 0.10 were excluded. If any pre-survey frequency of a sharing behavior reached significance in the model, all three were retained. All analyses were completed in SAS 9.4 (SAS Institute Inc., Cary, NC, USA).

## Results

The sample included 405 Hispanic and 413 non-Hispanic women. Demographic characteristics of the sample are given in Table 1. The non-Hispanic sample was 97.6% white or Caucasian.

**Table 1:** Demographics of Sample

	Hispanic *N* = 405	Non-Hispanic *N* = 413	Chi-Square *p*-value
	*n* (%)		
Mean Age in Year (SD)	28.34 (5.18)	29.41 (5.03)	0.003
Youngest Child at Home			
Less than a Year	110 (27.16)	89 (21.55)	0.29
1 Year old	99 (24.44)	121 (29.30)	
2 years old	75 (18.52)	78 (18.89)	
3 years old	60 (14.81)	55 (13.32)	
4 or 5 years old	61 (15.06)	70 (16.95)	
Highest Education Received			
High School Graduate, GED, or less	94 (23.21)	97 (23.49)	0.55
Some College or Associate Degree	201 (49.63)	191 (46.25)	
College Graduate or Post Graduate Degree	110 (27.16)	125 (30.27)	
Income as a Percentage of Federal Poverty Level			
< 100% FPL	98 (24.32)	80 (19.37)	0.06
100–200% FPL	139 (34.49)	131 (31.72)	
> 200% FPL	166 (41.19)	202 (48.91)	
Marital Status			0.002
Married or Member of unmarried couple	309 (76.30)	351 (84.99)	
Widowed, Divorced, Separated, Unmarried	96 (23.70)	62 (15.01)	
Pregnancy Status			0.9221
Not pregnant or planning a pregnancy	298 (73.58)	308 (74.58)	
Planning a pregnancy within 12 months	81 (20)	78 (18.89)	
Currently pregnant	26 (6.42)	27 (6.54)	
Crowding			<.0001
High	131 (32.35)	47 (11.38)	
Average/Low	274 (67.65)	366 (88.62)	

Hispanic and non-Hispanic women were similar with respect to many demographic characteristics. However, the average age of Hispanic women was slightly younger than non-Hispanic women, Hispanic women were less likely to be married or part of an unmarried couple and Hispanic women were more likely to report living in a home with high levels of crowding (32.4% vs. 11.4%; *p* < .0001). Among Hispanic women, 88% were born in the U.S. The majority of Hispanic women (59%) identified as Mexican, Mexican American, or Chicana while16.8% identified as Puerto Rican. The remaining 24% identified as “other” Hispanic or Latina origin. Among all Hispanics, 55% reported speaking mostly English in their homes, while 37% reported speaking English and Spanish equally in their homes.

Approximately 13% of the overall sample reported that they were very familiar or somewhat familiar with CMV. Hispanic women reported higher levels of CMV awareness (Hispanic 16.8% vs. non-Hispanic 9.7%; *p* = 0.002). Among women who reported familiarity with CMV (*n* = 108) Hispanic women had a higher, but non-significant, score on the knowledge scale (*M*_*Hispanic*_ = 4.40; *M*_*non-Hispanic*_ = 3.78; *p* = 0.23). Pre-survey participation in CMV risk behaviors is given in Table 2. Non-Hispanic women reported more frequent participation for two risk behaviors: sharing eating utensils and kissing a child on the lips, as compared to Hispanic women.

**Table 2:** Self-Reported pre-survey frequency of participation in select cytomegalovirus (CMV) risk behaviors^a^

	Hispanic	Non-Hispanic	*p*-value^b^
	*N* = 405	*N* = 413	
	Mean^c^ (95% CI)		*p*-value
Share the same cup with your child	2.67 [2.53, 2.81]	2.71 [2.58, 2.85]	0.65
Share eating utensils (fork or spoon) with your child	2.87 [2.73, 3.01]	3.08 [2.95, 3.22]	0.04
Share food with your child (take bites from the same food)	3.35 [3.21, 3.48]	3.50 [3.37, 3.63]	0.11
Kiss your child on their lips	3.27 [3.12, 3.42]	3.59 [3.44, 3.74]	0.004

^a^Respondent’s were asked to rate their frequency of participation in CMV risk behaviors using the following question stem: When your youngest child was in diapers how often did you do each of the following? (If your youngest child is still in diapers, think about what you currently do)

^b^*p*-values derived from a t-test for the difference in means between Hispanic and non-Hispanic respondents

^c^Frequency of behaviors were reported on a 5-point Likert scale where 1 = Never and 5 = every day. Higher mean values indicate greater frequency of participation in CMV risk behaviors

**Fig. 1:**
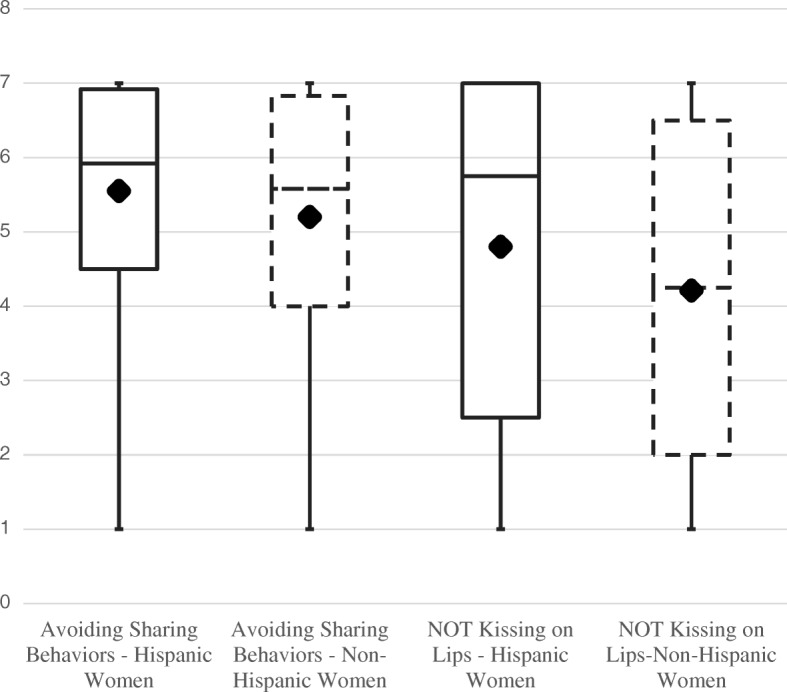
Hispanic and non-Hispanic women’s attitudes toward not kissing on lips and avoiding sharing behaviors. Attitudes toward CMV prevention behaviors were assessed by asking women to rate on four 7-point semantic differential scales: impractical (1) to practical (7), inconvenient (1) to convenient (7), difficult (1) to easy (7) and unrealistic (1) to realistic (7) for each of the CMV prevention behaviors. Higher values indicate that women viewed the behaviors are more practical, more convenient, easier and more realistic. Overall attitudes toward kissing on the lips was calculated as the average of the four adjectives. Overall attitude for the sharing behaviors (sharing a cup, sharing eating utensils, and sharing food with a child) were calculated into a single scale as the average of the four adjectives. Box plots summarize the distribution of attitudes across the behaviors: median (diamond shape), interquartile range (25th and 75th percentiles), and range (minimum and maximum)

Both Hispanic and non-Hispanic women reported generally positive attitudes toward CMV prevention behaviors (see Fig. 1 and Additional file 1). However, Hispanic women reported more positive attitudes than non-Hispanic women. The mean attitude score for avoiding sharing behaviors was 5.55 for Hispanic women compared to 5.20 in non-Hispanic women (*p* = 0.002). Similarly, Hispanic women had a mean score of 4.80 for attitudes toward avoiding kissing a child on the lips, compared to 4.21 in non-Hispanic women (*p* = .0002). For both groups, attitudes were significantly lower toward avoiding kissing than attitudes toward avoiding sharing behaviors (*p* < .0001).

Among Hispanic women, U.S. born Hispanics reported kissing their child on the lips (*M*_*U.SBorn*_ *=* 3.35 vs. *M*_*BornOutsideU.S*_*. =* 2.65; *p* = 0.005) and sharing food, cups and eating utensils (*M*_*U.SBorn*_ *=* 3.05 vs. *M*_*BornOutsideU.S*_*. =* 2.62; *p* = 0.03) more frequently than did Hispanic women born outside of the U.S. Hispanic women who reported speaking mostly Spanish in their homes also reported lower frequency of kissing a child on the lips (*M*_*Spanish*_ *=* 2.65, *M*_*English&Spanish*_ *=* 2.97, *M*_*English*_*. =* 3.56; *p*= .0005) compared to Hispanic women who spoke mostly English or English and Spanish equally at home. Similarly, those would spoke mostly Spanish at home had more positive attitudes toward avoiding kissing children on the lips than those who spoke English mostly, or English and Spanish equally (*M*_*Spanish*_ *=* 5.92, *M*_*English&Spanish*_ *=* 5.45, *M*_*English*_*. =* 4.20; *p* < .0001) and reported higher perceived behavioral control (*M*_*Spanish*_ *=* 6.05, *M*_*English&Spanish*_ *=* 5.37, *M*_*English*_*. =* 4.81; *p* = .0005).

Women of both groups reported high mean levels of perceived behavioral control for avoiding sharing food, cups, and utensils with their children (*M*_*Hispanic*_ = 5.72; *M*_*non-Hispanic*_ = 5.64; *p* = 0.40). Perceived behavioral control for not kissing a child on the lips was lower than for sharing behaviors in both groups. However, Hispanic women (*M* = 5.11) reported significantly higher perceived behavioral control for avoiding kissing on the lips than non-Hispanic women (*M* = 4.63; *p* = 0.001).

There were differences in how the two groups of women showed affection to their children. The majority of women in the sample reported that they kissed, hugged or held their children to show affection “always.” An estimated 84.4% of Hispanic women reported they “always” hugged their children to show affection compared to 75.5% of non-Hispanic women (*p* = 0.004). Similarly, a higher percentage of Hispanic women kissed (72.4% vs. 56.9%; *p* < .0001) and held (71.1% vs. 55.2%; *p* < .0001) their children “always” as compared to non-Hispanic women. The question about how affection was expressed asked about kissing in general, and not about the location of the kiss.

For both groups of women, the plurality reported that they neither agreed nor disagreed that that people important to them and whose opinions they valued thought they should share food, cups, and utensils (Table 3).

**Table 3:** Subjective norms for select CMV risk behaviors in Hispanic and non-Hispanic women

	Hispanic *N* = 413	Non-Hispanic *N* = 405	*p*-value^a^	Hispanic *N* = 413	Non-Hispanic *N* = 405	*p*-value^a^
	People who are important to me think I should:			People whose opinion I value think I should:		
	*n* (%)	*n* (%)		*n* (%)	*n* (%)	
Share the same cup with my child						
Strongly agree	24 (5.93)	20 (4.84)	0.02	32 (7.9)	17 (4.12)	<.0001
Somewhat agree	58 (14.32)	43 (10.41)		56 (13.83)	39 (9.44)	
Neither agree nor disagree	162 (40.00)	207 (50.12)		150 (37.04)	214 (51.85)	
Somewhat disagree	47 (11.60)	54 (13.08)		49 (12.10)	58 (14.04)	
Strongly disagree	114 (28.15)	89 (21.55)		118 (29.14)	85 (20.58)	
Share eating utensils			0.19			
Strongly agree	24 (5.94)	22 (5.33)		34 (8.40)	20 (4.84)	0.003
Somewhat agree	61 (15.10)	49 (11.86)		52 (12.84)	49 (11.86)	
Neither agree nor disagree	164 (40.59)	200 (48.43)		153 (37.78)	210 (50.85)	
Somewhat disagree	55 (13.61)	57 (13.8)		63 (15.56)	51 (12.35)	
Strongly disagree	100 (24.75)	85 (20.58)		103 (25.43)	83 (20.10)	
Share food with my child			0.04			
Strongly agree	39 (9.63)	37 (8.96)		43 (10.62)	28 (6.78)	0.02
Somewhat agree	88 (21.73)	66 (15.98)		79 (19.51)	73 (17.68)	
Neither agree nor disagree	159 (39.26)	198 (47.94)		155 (38.27)	197 (47.7)	
Somewhat disagree	37 (9.14)	46 (11.14)		41 (10.12)	49 (11.86)	
Strongly disagree	82 (20.25)	66 (15.98)		87 (21.48)	66 (15.98)	
Kiss my child on the lips			<.0001			
Strongly agree	70 (17.28)	77 (18.64)		69 (17.04)	72 (17.43)	<.0001
Somewhat agree	54 (13.33)	71 (17.19)		50 (12.35)	86 (20.82)	
Neither agree nor disagree	131 (32.35)	166 (40.19)		137 (33.83)	154 (37.29)	
Somewhat disagree	38 (9.38)	43 (10.41)		31 (7.65)	38 (9.20)	
Strongly disagree	112 (27.65)	56 (13.56)		118 (29.14)	63 (15.25)	

^a^*p*-value derived from a chi-square test for the difference of proportions for Hispanic vs. non-Hispanic women

Results of the regression models are given in Table 4. Increased frequency of sharing food, cups, or utensils was associated with having children between ages 1–5, having a high school education or less increasing maternal age, being pregnant or planning a pregnancy, increased perception that others expected sharing behaviors (subjective norms) and higher levels of parental affection. Similarly, increased frequency of kissing a child on the lips was associated with increased subjective norms and increased parental affection. For both avoiding sharing behaviors and avoiding kissing a child on the lips, increased perception that others expected the behaviors and increased frequency of performing the risk behaviors were associated with decreased perceived behavioral control.

**Table 4:** Factors associated with CMV risk behaviors, perceived behavior control and attitudes toward CMV prevention behaviors

	Model 1	Model 2	Model 3	Model 4	Model 5	Model 6
Variable	Frequency of Sharing	Frequency of Kissing	Perceived Behavioral Control: NOT Sharing	Perceived Behavioral Control: NOT Kissing	Attitudes toward NOT sharing	Attitudes toward NOT Kissing on the Lips
	β (SE)					
Hispanic					0.20 (0.08)^*^	
Familiar with CMV					0.22 (0.12)	0.32 (0.15)^*^
Age of youngest child at home						
4–5 years	0.47 (0.13)^**^				0.46 (0.13)^**^	
3 years	0.60 (0.13)^***^				0.14 (0.14)	
2 years	0.57 (0.12)^***^				0.08 (0.13)	
1 year	0.82 (0.11)^***^				−0.09 (−0.12)	
< 1 year	Ref				Ref	
Education						
≤ High School Graduate	0.35 (0.11)^*^				0.24 (0.12)^*^	
Some College	0.13 (0.09)				0.08 (0.10)	
College Graduate or Above	Ref				Ref	
Pregnant or Planning a Pregnancy	0.18 (0.09)^*^					
Mother’s Age in Years	0.02 (0.001)^*^					
Marital Status						
Married/Unmarried Couple		0.19 (0.11)	0.27 (.06, .49)			
Never Married/Widowed/Separated/Divorced		Ref	Ref			
Household Crowding						
High Crowding					0.21 (0.10)*	0.27 (0.12)^*^
Average or Low Crowding					Ref	Ref
Subjective Norms for Sharing Behaviors	0.47 (0.04)^***^		−0.33 (0.05)^***^		−0.24 (0.04)^***^	
Subjective Norms for Kissing on Lips		0.67 (0.04)^***^		−0.38 (0.05)^***^		−0.25 (0.05)^***^
Parental Affection	0.29 (0.9)^**^	0.44 (0.10)^***^				
Perceived Behavioral Control for Avoiding Sharing Behaviors					0.55 (0.3)^***^	
Perceived Behavioral Control for Avoiding Kissing on the Lips						0.53 (0.03)^***^
Frequency of Sharing Food			−0.14 (0.05)^*^		−0.05 (−0.05)	
Frequency of Sharing a Cup			−0.06 (0.05)		−0.13 (− 0.04)^*^	
Frequency of Sharing Utensils			−0.11 (0.05)^*^		−0.11 (− 0.05)^*^	
Frequency of Kissing a Child on the Lips				−0.59 (0.04)^***^		−0.42 (0.04)^***^
r^2^	0.25	0.32	0.20	0.39	0.49	0.60

*p < .05; **p < .01;***p < .0001

More positive attitudes toward avoiding sharing food, cups, and utensils with a child were significantly associated with Hispanic ethnicity, having an older (4 to 5 year-old) child, a high school education, higher levels of perceived behavioral control, and above average household crowding. Additionally, less positive attitudes toward avoiding sharing behaviors were associated with higher levels of subjective norms and increased pre-survey frequency of sharing cups or utensils. Nearly half of the variation in attitudes toward sharing behaviors was explained by the final model (r^2^ = 0.49). Attitudes toward not kissing a child on the lips showed a similar pattern, familiarity with CMV was associated with an increase in positive attitudes toward behavior change as were increased perceived behavioral control and higher household crowding. Conversely, increased pre-survey frequency of kissing a child on the lips and increased perception that others expected that they kiss a child on the lips were associated with less positive attitudes toward behavior change. Most the variation (r^2^ = 0.60) in attitudes toward avoiding kissing a child on the lips was explained by the final model (model #6).

## Discussion

The purpose of this research was to understand Hispanic and non-Hispanic women’s attitudes toward CMV prevention behavior. We also examined the association between attitudes, subjective norms and perceived behavioral control. This is the first research that has focused specifically on Hispanic women. Hispanic women reported engaging in CMV risk behaviors less often than non-Hispanic women. There were also between-group differences in attitudes, perceived behavioral control and subjective norms. Ethnicity was a significant predictor in only one of six regression models indicating that other factors, like subjective norms and perceived behavioral control are more important in predicting attitudes than ethnicity.

CMV awareness rates in both groups were similar to other research [13, 17]. Similarly, low rates of CMV knowledge, of transmission modes, and the frequency of participating in CMV prevention and risk behaviors was similar to other research that used a national sample of women [13, 16, 17].

Although CMV prevalence is higher among Hispanics than non-Hispanics, certain behavioral risk factors for CMV may be less common among Hispanic women as was found in the present study. Similarly, a study comparing the prevalence of risk factors for oral bacteria transmission observed that Hispanic parents were less likely to kiss their children on the lips, share food and utensils with their children, and put their toddler’s pacifier in their own mouth [24]. However, other researchers have suggested that differences in infection rates between ethnic groups may be due to household crowding [19]. In our study, Hispanic women were living in higher density housing than non-Hispanic women.

For CMV behavior modification to occur, a woman must believe that she is in control of performing the recommended behavior [25]. Women reported high levels of perceived behavioral control for avoiding sharing behaviors; scores were somewhat lower for not kissing a child on the lips. These levels of perceived behavioral control are similar to other research we have conducted [17].

In the present study, Hispanic women reported kissing to show affection more often than non-Hispanic women, yet they reported kissing on the lips less often than non-Hispanic women. This was particularly true for Hispanic women who were foreign born or reported speaking mostly Spanish in their homes. Being foreign born and/or speaking primarily Spanish in the home may indicate less acculturation. This tendency to be more expressive may be based in culture, as Hispanic women are more apt to engage in more frequent and close touch [26]. A study of European Americans and Mexican Americans showed that persons who identified as less acculturated and more connected to their heritage were more comfortable with affectionate touch [27]. Though that study did not assess kissing specifically it suggests that Hispanic culture is more demonstrative than other Anglo/European cultures. Though in the present study Hispanic women respondents reported being more demonstrative to their child, they believed they had more control to not kiss on the lips than did non-Hispanic white women. Again, this may represent cultural differences in way affection is expressed and is consistent with our finding that Hispanic women kiss their children on the lips less frequently.

Women with more positive attitudes toward the CMV prevention behaviors also were more confident in their ability to not share cups, food or utensils, and to not kiss on the lips. Although both groups of women held generally positive attitudes toward the CMV prevention behaviors, scores were lower for not kissing a child on the lips. The tendency to have less positive attitudes toward not kissing on the lips than other prevention behaviors is similar to past research completed with a U.S. panel of women [17]. Overall, there are between group differences as Hispanic women have more positive attitudes than non-Hispanic toward the CMV prevention behaviors. This may be because they reportedly do these behaviors less often than non-Hispanic women do. Women who perceived that others expected them to engage in kissing on the lips and sharing behaviors had decreased attitudes scores. The final regression models explained a substantial amount of the variation in attitudes toward the CMV prevention behaviors.

It appears that women more frequently engaged in kissing on the lips and the sharing behaviors when they felt a social expectation to do so. Each culture has a “social code” for how an adult is expected to behave in relation to their child [28]. In this study, an increased social expectation was also associated with lower levels of perceived behavior control. Women may feel that it is difficult to believe they can change a behavior when they do the behavior frequently and perceive that others expect them to do it as well. Although subjective norms were influential, there was a moderate proportion of women who neither agreed nor disagreed with the subjective norms statements. It could be that these CMV prevention behaviors are not frequently discussed verbally, but rather acceptable behavior is assumed.

### Limitations

This is a cross-sectional descriptive study with self-report data, which may be subject to recall bias and social desirability bias. Though we did not provide any CMV-related educational material about the behaviors that increase CMV risk, it is possible that the wording of the questions implied the preferred behavior. Therefore, it is probable that all responses reflect a degree of social desirability bias. The difference between Hispanic and non-Hispanic women’s responses might be attributable to cultural differences in the influence of social desirability. Research has shown that responses vary by culture and Hispanics tend to be influenced more by social desirability [29–31].

This is national sample using an on-line panel. The data may not reflect all women of childbearing years. However, our demographic data are similar to other national studies of CMV awareness and attitudes [9, 16]. The measure of attitudes was developed by the authors and has been used in prior research. While reliability estimates are available, validity evidence is not. Finally, this survey was taken in English, 88% of the sample was born in the U.S. and therefore may not represent all Hispanic women, particularly those who have recently come to the U.S. or are not fluent in English. As Hispanic women are at increased risk of congenital CMV infection, additional research should consider the inclusion of recent immigrants and non-English speaking Hispanics.

## Conclusion

Research has shown that communicating with women to reduce CMV infection can be effective [11, 32]. In the current study, women expressed positive attitudes toward the CMV prevention behaviors and feel that they can do them. These data suggest that while there may be ethnic differences in attitudes toward CMV prevention behaviors between Hispanic women and non-Hispanic women of childbearing age, that subjective norms and perceived behavior control are more important predictors of attitudes than ethnicity. In developing strategic efforts to increase women’s participation in CMV prevention behaviors, a focus on understanding what is influencing subjective norms and feelings of control over the behavior, may be warranted.

## Additional file

Additional file 1: Hispanic & non-Hispanic women’s attitudes toward individual CMV prevention behaviors. Table that shows Hispanic and non-Hispanic women’s attitudes toward four CMV prevention behaviors. (DOCX 16 kb)

## Abbreviations

CMV: Cytomegalovirus
FPL: Federal poverty level
