# Supplementary material for: Attitudes of U.S. Hispanic and non-Hispanic women toward congenital CMV prevention behaviors: a cross sectional study
Source: BMC Pregnancy Childbirth. 2018 May 24;18:181. doi: 10.1186/s12884-018-1807-0 (PMC5968502; doi:10.1186/s12884-018-1807-0)
Supplement: Supplementary file 1 — Hispanic & non-Hispanic women’s attitudes toward individual CMV prevention behaviors. Table that shows Hispanic and non-Hispanic women’s attitudes toward four CMV prevention behaviors. (DOCX 16 kb) [file 12884_2018_1807_MOESM1_ESM.docx]

**Additional File 1.**

**Hispanic & non-Hispanic women’s attitudes toward individual CMV prevention behaviors**

| CMV Prevention Behavior |  | Impractical to Practical | Inconvenient to Convenient | Difficult to Easy | Unrealistic to Realistic |
| --- | --- | --- | --- | --- | --- |
|  |  | Mean [95% CI] | | | |
| NOT share the same cup with my child | Hispanic | 5.65 [5.48, 5.82] | 5.36 [5.18, 5.55] | 5.73 [5.56, 5.90] | 5.68 [5.50, 5.85] |
|  | Non-Hispanic | 5.42 [5.25, 5.60] | 5.20 [5.01, 5.38] | 5.38 [5.20, 5.57] | 5.36 [5.17, 5.55] |
|  | p-value | 0.07 | 0.21 | 0.008 | 0.02 |
|  |  |  |  |  |  |
| NOT share eating utensils with my child | Hispanic | 5.72 [5.55, 5.89] | 5.46 [5.28, 5.65] | 5.68 [5.51, 5.86] | 5.74 [5.57, 5.91] |
|  | Non-Hispanic | 5.37 [5.19, 5.55] | 5.20 [5.01, 5.39] | 5.40 [5.22, 5.59] | 5.38 [5.19, 5.56] |
|  | p-value | 0.006 | 0.05 | 0.03 | 0.004 |
|  |  |  |  |  |  |
| NOT share food with my child | Hispanic | 5.51 [5.34, 5.68] | 5.22 [5.03, 5.40] | 5.45 [5.27, 5.63] | 5.43 [5.25, 5.61] |
|  | Non-Hispanic | 5.03 [4.84, 5.23] | 4.79 [4.58, 4.99] | 5.02 [4.82, 5.22] | 4.89 [4.69, 5.09] |
|  | p-value | 0.0003 | 0.002 | 0.002 | <.0001 |
|  |  |  |  |  |  |
| NOT kiss my child on their lips | Hispanic | 4.98 [4.75, 5.20] | 4.85 [4.62, 5.08] | 4.65 [4.40, 4.89] | 4.70 [4.45, 4.94] |
|  | Non-Hispanic | 4.37 [4.14, 4.59] | 4.39 [4.17, 4.61] | 4.00 [3.76, 4.23] | 4.08 [3.85, 4.31] |
|  | p-value | 0.0002 | 0.0043 | 0.0002 | 0.0003 |

Note: Attitudes toward CMV prevention behaviors were assessed by asking women to rate on four 7-point semantic differential scales: impractical (1) to practical (7), inconvenient (1) to convenient (7), difficult (1) to easy (7) and unrealistic (1) to realistic (7) for each of the CMV prevention behaviors. Higher values indicate that women viewed the behaviors are more practical, more convenient, easier and more realistic.
